# Spatial transcriptome profiling of normal human liver

**DOI:** 10.1038/s41597-022-01676-w

**Published:** 2022-10-19

**Authors:** Shizhe Yu, Haoren Wang, Lingpeng Yang, Yingxue Yan, Qiang Cai, Duo Ma, Long Jiang, Zehai Gao, Zhiyong Yu, Zongping Xia

**Affiliations:** 1 Clinical Systems Biology Laboratories, Translational Medicine Center, The First Affiliated Hospital of Zhengzhou University, Zhengzhou, 450052 Henan China; 2 Department of Hepatobiliary Surgery, The Affiliated Hospital of Yunnan University, Kunming, 650021 Yunnan China

**Keywords:** Liver, Data acquisition, Single-cell imaging

## Abstract

The comprehensive study of the spatial-cellular anatomy of the human liver is critical to addressing the cellular origins of liver disease. Here we conducted spatial transcriptomics on normal human liver tissue sections, providing detailed information of liver zonation at the transcriptional level. We present 6581 high-quality spots from normal livers of two human donors. In this dataset, cells were mainly hepatocytes, and we classified them into four sub-groups. Collectively, these data provide a reliable reference for studies on spatial heterogeneity of liver lobules.

## Abstract (summary)

**Table:** 

Measurement(s)	mRNA Expression
Technology Type(s)	spatial transcriptomics
Sample Characteristic - Organism	Homo sapiens

## Background & Summary

The liver is a critical multifunctional organ, serving as a central coordinator for metabolic homeostasis and contributing to the eradication of various xenobiotic compounds and toxins^1^. The ability to manipulate multi-tasks for the liver mainly depends on the spatial zonation, which is based on the highly structured repeating anatomical units termed liver lobules^2^. Owing to the blood flow and morphogens, hepatocytes along the lobular axis, which is from portal veins to central veins, are exposed to different physicochemical environments, resulting in differential expression profiles with a further tendency to distinct functions^3–5^. On the basis of hepatocytic differences, liver lobules are divided into three zones, zone 1–3 from portal veins to central veins, with various essential liver functions^6^.

In the 20th century, researchers used several technologies to explore liver zonation characteristics, including *in-situ* hybridization, immunohistochemistry, and microdissection combined with transcriptome sequencing^7–9^. However, the precision and depth of these studies are limited.

Nowadays, single-cell RNA sequencing makes it possible to measure the genome-scale information with a more increased resolution and has revealed that 50% of hepatocyte genes are expressed in a zonation manner^10–12^. Nevertheless, dissociating tissue into single-cell suspensions loses inherent spatial information, which can not be reconstructed in silico completely^13,14^. Moreover, on reviewing the literature, the proportion of hepatocytes was less than what should be expected^15–19^. Such bias may be due to the size of hepatocytes and their intolerance to tissue dissociation methods.

To address these issues, we conducted spatial transcriptomics on the normal human liver with 10X Genomics Visium technology and obtained 6581 high-quality human liver spots from two organ donors (liver 1 and 2). Consistent with the proportion in normal liver, cells in these data were largely hepatocytes^20^.They were divided into four subgroups along the lobular axis with the combination of unbiased classification and location of spots. The data contain detailed spatial information of normal liver, providing a reliable reference for liver disease research.

## Methods

**Fig. 1:**
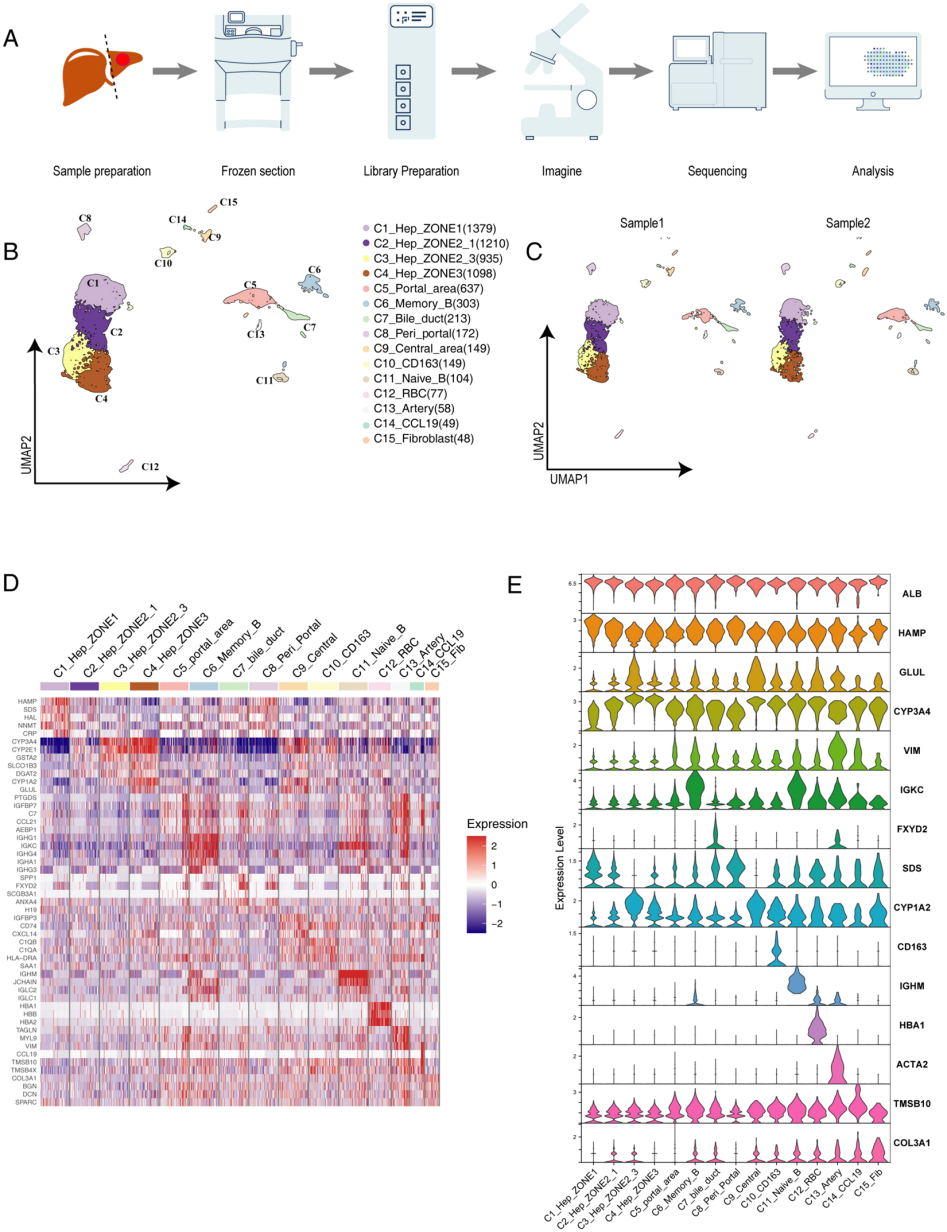
Spatial transcriptome reveals the cell populations of the normal human liver. (**A**) Overview of the Visium process using human liver tissue samples. (**B**) Uniform manifold approximation and projection (UMAP) plot showing the unbiased classification of liver cells. (**C**) Split UMAP plot showing the batch effect between the two different liver samples. (**D**) Heatmap showing the marker genes of each cluster, highlighting the top marker genes for each cluster. (**E**) Violin plot illustrating the selected marker genes of each cluster.

We introduce a summary of the liver spatial transcriptome method. The whole procedure included the acquisition of human liver tissue, preparing frozen sections, and Visium sample processing (Fig. 1A).

### Ethical approval

We received approval from the Ethics Committee of the Affiliated Hospital of Yunnan University, and signed informed consent was obtained from all patients.

### Human liver tissue procurement

Fresh human liver samples (Supplementary Table S1) were collected at the Affiliated Hospital of Yunnan University. Samples were obtained from patients undergoing partial liver resection for hepatic hemangioma. Normal liver tissues were obtained at least 1 cm away from hemangioma, and the HE images support the normal liver diagnosis(Supplementary Figure 1A,B).

First, fresh samples were obtained from the operating theatre and immediately rinsed three times using pre-cooled sterile saline to wash away any residual blood and red blood cells on the surface. The surface was blotted dry using sterile gauze, the whole process taking about 1 min.

A small amount of pre-cooled optimal cutting temperature (OCT) compound (Sakura Finetek, Torrance, CA) was first added to cover the bottom in a 7 mm*7 mm embedding mold, followed by gentle clamping of the liver tissue block with forceps and placing it in the mold. Sufficient OCT was then added to completely cover the specimen, taking care to prevent the appearance of air bubbles. The whole process was completed on ice.

The tissue blocks were then immediately placed in dry ice boxes for quick-frozen storage and transferred to a deep cryogenic refrigerator for storage. Subsequent sample quality control and tissue optimization processes were carried out.

### Frozen sections preparation and quality control

The liver tissue block was mounted onto the specimen disc of a cryostat (Leica Microsystems) using OCT. The cryomicrotome was precooled to −20 °C, and the liver tissue block was mounted onto the specimen disc using OCT compound. Frozen sections were cut into 10 μm thick. 3–5 pieces of sections were collected into pre-cooled 1.5 ml centrifuge tubes for total-RNA extraction.

The RNAeasy™ Animal RNA Isolation Kit with Spin Column (R0024; Beyotime, Shanghai, China) was used to obtain total RNA from frozen sections according to the manufacturer’s guidelines. RNA concentration was measured by nanodrop (ThermoFisher Scientific, MA, USA) and RNA quality was assessed by Qsep1 bioanalyzer (BiOptic Inc., Taiwan, China). Samples with RNA integrity number (RIN) above 8.0 were used for sequencing.

### Tissue Optimization

Visium spatial tissue optimization (TO) slides contain eight mRNA capture areas with oligonucleotides, and each capture area is defined by an etched frame. Prior to library preparation, the Visium Spatial Tissue Optimization workflow allows the user to optimize permeabilization conditions for a tissue of interest.

10 μm tissue sections from the same sample were placed onto the Capture Areas of the TO slides. These sections fixed and stained, as described in Tissue Fixation & Staining Demonstrated Protocols (CG000238 Rev D), and then permeabilized for 4 min, 8 min, 12 min, 16 min, 20 min, 24 min, 28 min, respectively. The mRNA released during permeabilization bound to capture probes on the slide, and fluorescently labeled nucleotides were used to visualize synthesized cDNA. Tissue was enzymatically removed, leaving fluorescent cDNA covalently linked to oligonucleotides on the TO slide. Fluorescent cDNA and bright-field images were scanned by a 3DHISTECH Pannoramic Midi scanner (3D HISTECH Ltd., Budapest, Hungary). The permeabilization time that results in maximum fluorescence signal with the lowest signal diffusion is optimal. Thus we chose 8 min for our final permeabilization time.

### Visium gateway gene expression process

The Visium Gateway Gene Expression Slide includes 2 Capture Areas (6.5 × 6.5 mm). The Capture Area has ~5,000 gene expression spots. 10 μm tissue sections from the 2 samples were placed onto the Capture Areas of the Visium slides. Tissue sections placed on these Capture Areas were fixed, stained, imaged and permeabilized as described in the previous step, and cellular mRNA was captured by the primers on the gene expression spots. Second Strand Mix was added to the tissue sections on the slide to initiate second strand synthesis. The 10x Genomics Visium Spatial Gene Expression Reagent Kits user guide (CG000239 Rev D) was followed to prepare the spatial transcriptome library. All the cDNA generated from mRNA captured by primers on a specific spot shared a common Spatial Barcode. The cDNA concentration was detected by a Qubit4.0 fluorometer (Invitrogen). Libraries were generated from the cDNA and sequenced and the Spatial Barcodes were used to associate the reads back to the tissue section images for spatial gene expression mapping.

### The sequencing process

Libraries were loaded at 300 pM and sequenced on a NovaSeq 6000 System (Illumina) using a NovaSeq S4 Reagent Kit (200 cycles, 20027466, Illumina), at a sequencing depth of approximately 250–400 M read-pairs per sample. Sequencing was performed using the following read protocol: read 1, 28 cycles; i7 index read, 10 cycles; i5 index read, 10 cycles; read 2, 91 cycles.

### Visium raw data processing

Raw FASTQ files (Supplementary Table S2) and histology images were processed with the Space Ranger software version 1.3.1, which uses STAR v.2.5.1b for genome alignment^21^, against the Cell Ranger hg38 reference genome “refdata-cellranger-GRCh38-3.0.0”, available at: (http://cf.10xgenomics.com/supp/cell-exp/refdata-cellranger-GRCh38-3.0.0.tar.gz).

### Use of STutility for quality control (QC) and second data analysis after correction of batch effect

**Fig. 2:**
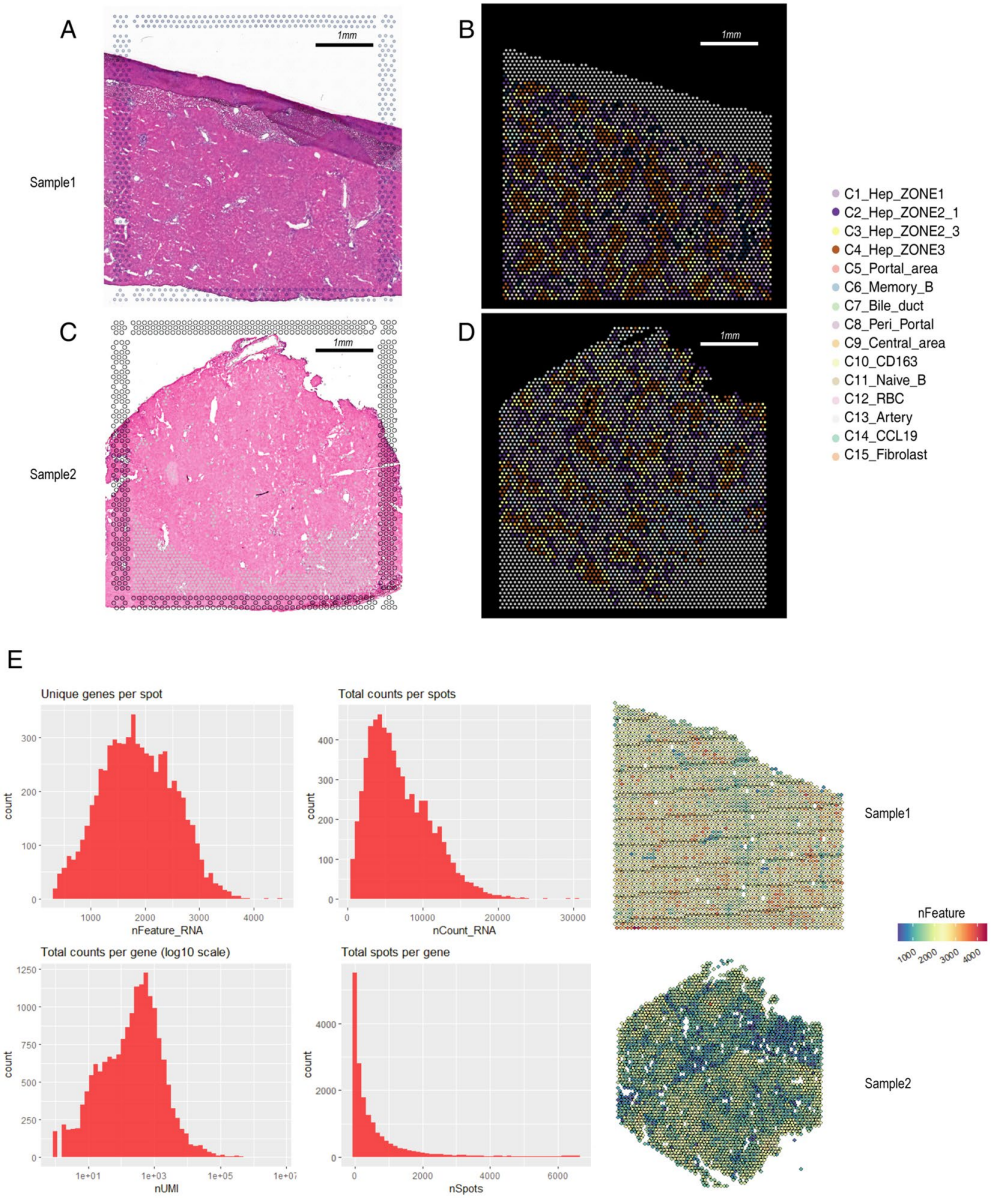
Quality control (QC) of human liver spatial transcriptome data. (**A**,**C**) HE staining of the 2 samples. (**B**, **D**) Visium Plots showing the spatial location of each cluster. (**E**) Bar and Feature plots illustrating the number of genes, unique molecular identifiers (UMIs), and total counts of 2 liver samples in each spot.

We used the R (version 4.1.2, https://www.r-project.org/) and STutility R package (https://github.com/jbergenstrahle/STUtility). We used the “MergeSTData” function to merge the two liver datasets. According to the median number of genes in the liver samples, spots with <200 genes were filtered. As the spatial transcriptome did not capture doublet cells and “soup-RNA,” we did not set an upper limit on the threshold. After QC, 6581 high-quality liver spots were obtained. The relationship between the mRNA reads and the number of mRNAs was detected and visualized (Fig. 2E).

Genes were normalized using the Sctransform package, and high-variable genes (n = 3000) were conducted to the following principal-component analysis (PCA)^22^. The harmony package was used to correct the batch effect between the two samples^23^. 30 corrected PCs were selected as the input for uniform manifold approximation and projection (UMAP). We detected the batch effect between the two different liver samples (Fig. 1C). With a resolution of 0.5, spots were clustered by the “FindClusters” function and classified into 15 different spot types. Next, we used the “FindAllMarkers” function to find differentially expressed genes between each type of spots (Fig. 1D,E, Supplementary Table S3). Markers used to define cell types included HAMP, GLUL, CYP3A4, VIM, IGKC, SPP1, CYP1A2, CD163, IGHM, HBA1, ACTA2, and COL3A1. Since the Visium technology obtained a mixed transcriptome of multiple cells in a spot, we defined three specific regions (C5, C8, C9) in combination with spatial locations (Supplementary Figure 1D).

### Immunohistochemical validation

All immunohistochemical images were adapted from the Human Protein Atlas^24^.

## Data Records

Raw sequencing data have been deposited into the NCBI sequence read archive (SRA) database under accession number PRJNA821403^25^. All processed data have been uploaded to the figshare database^26^. These data include filtered_feature_bc_matrix.h5, cloupe file and spatial files.

## Technical Validation

The liver specimens were collected freshly dissected from organ donors, one 57 years-old male and one 39 years-old female. (Supplementary Table S1, Methods). The median genes per spot were 1980, higher than that from previous scRNA-seq dataset^10,18^ (Fig. 2E).

After QC, 6581 high-quality spots were further analyzed. According to the marker genes, cells were classified into 15 clusters that contained spots in the range of 48–1379 spots per cluster, corresponding to hepatocytes, biliary epithelial cells, liver endothelial cells, B cells and *et al*. (Fig. 1B,D). The clusters were visualized using UMAP, precisely matching with the HE staining results (Fig. 2A,C).

**Fig. 3:**
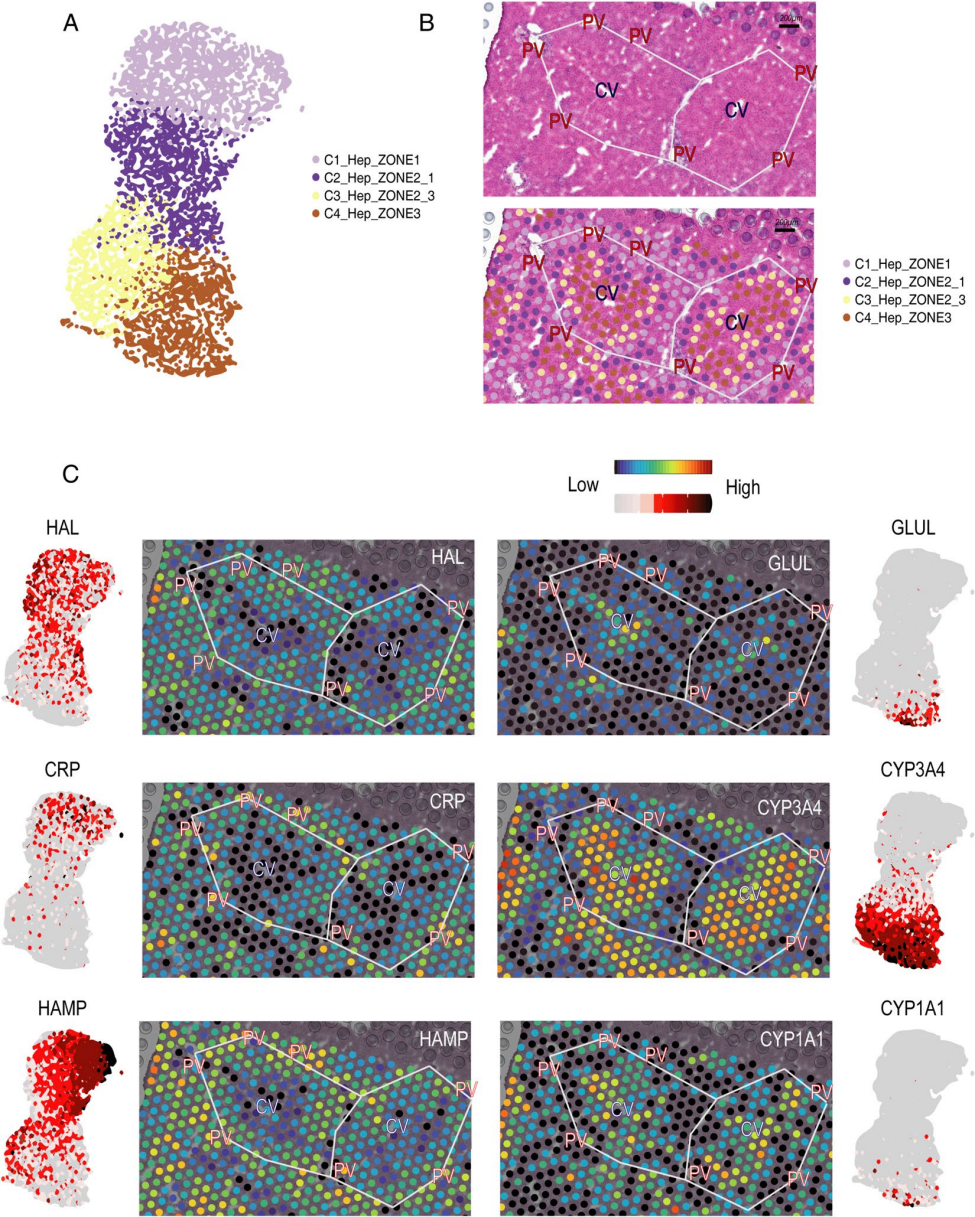
Spatial transcriptome revealing the zonation distribution of hepatocytes. (**A**) UMAP plot showing the fine clusters of hepatocytes. (**B**) HE and Visium Plots showing the zonation of the hepatocytes. (**C**) Visium and UMAP plots showing the expression levels of each gene on the tissue.

**Fig. 4:**
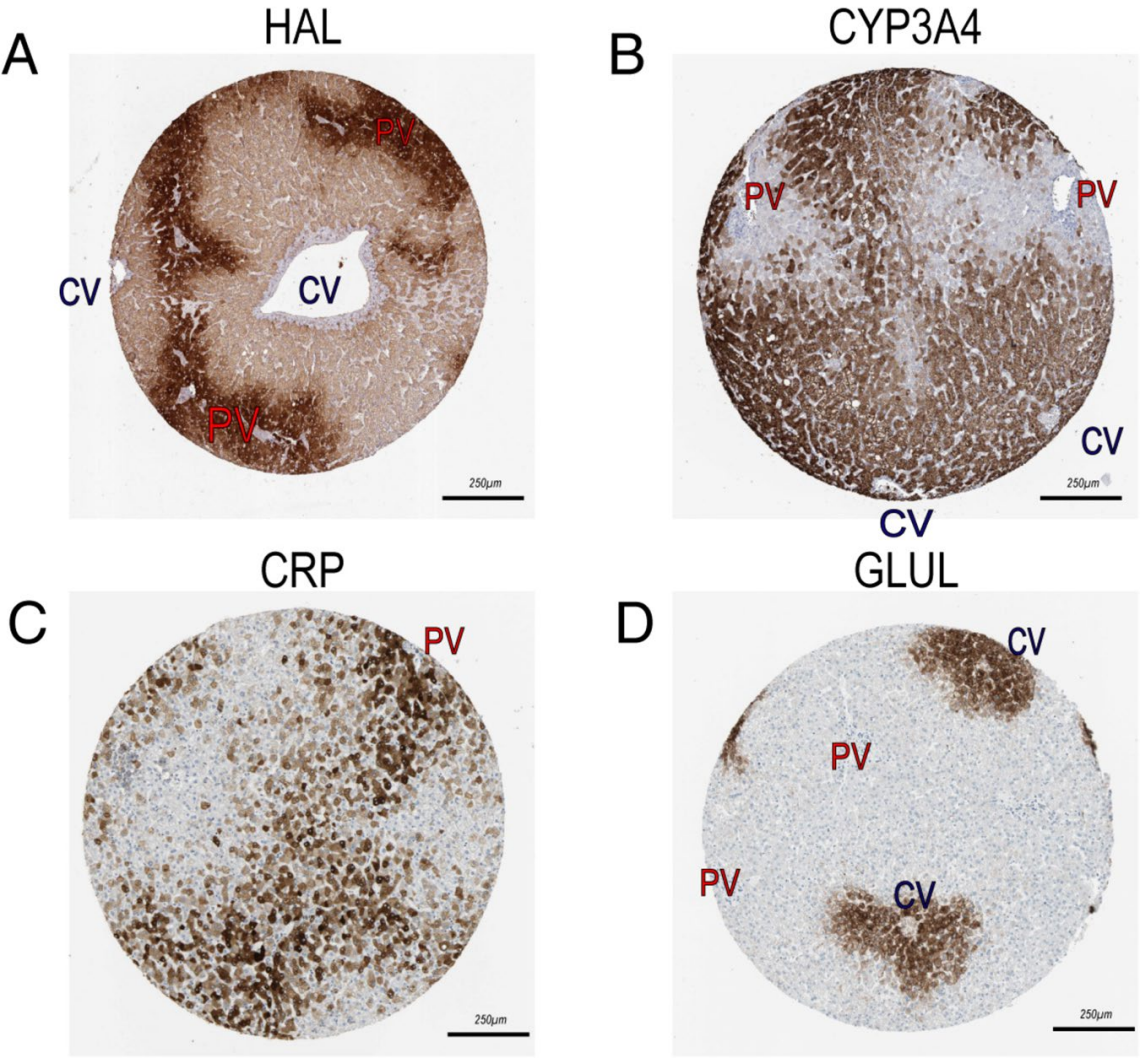
Representative IHC plots verifying the zonal distribution of differential genes at the protein level.

The results showed that most cells were hepatocytes, consistent with the natural condition. The hepatocytes were extracted and classified into four  sub-clusters. The embeddings of these four sub-clusters on the UMAP were precisely along the lobular axis, thus naming them as Zone 1 (near the portal veins), Zone 2-1, Zone 2-3, Zone 3 (near the central veins), respectively (Fig. 3A). To visualize cells in different zonation with Visium, we determined the region of portal veins and central veins with the help of HE staining and delimited the border of liver lobules (Fig. 2B,D). The differential genes related to the liver zonation, including the peri-portal area genes HAL, HAMP, and CRP and the peri-central area genes GLUL, CYP3A4, and CYP1A1, were located (Fig. 3 C). The IHC staining figures from the human protein atlas further confirmed these results (Fig. 4A–D). In sum, we contributed a reliable spatial transcriptomics atlas, benefiting the study of liver heterogeneity and providing a reference for spatial research of the liver disease.

## Supplementary information

Supplementary Table 1

Supplementary Table 2

Supplementary Table 3

Supplementary Figure 1

Supplementary Figure 2

Supplementary Figure 3

## Data Availability

The R code used in the analysis of the Visium data is available on GitHub (https://github.com/yuGithuuub/Normal_liver_visium). This R code is also available at figshare^26^.
