# Supplementary figures and images for: Spatial transcriptome profiling of normal human liver
Source: Sci Data. 2022 Oct 19;9:633. doi: 10.1038/s41597-022-01676-w (PMC9581974; doi:10.1038/s41597-022-01676-w)

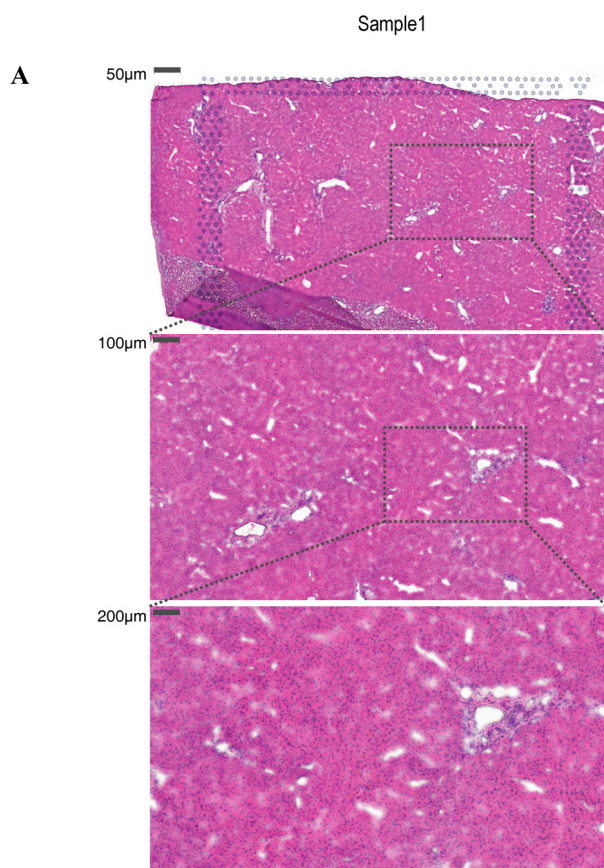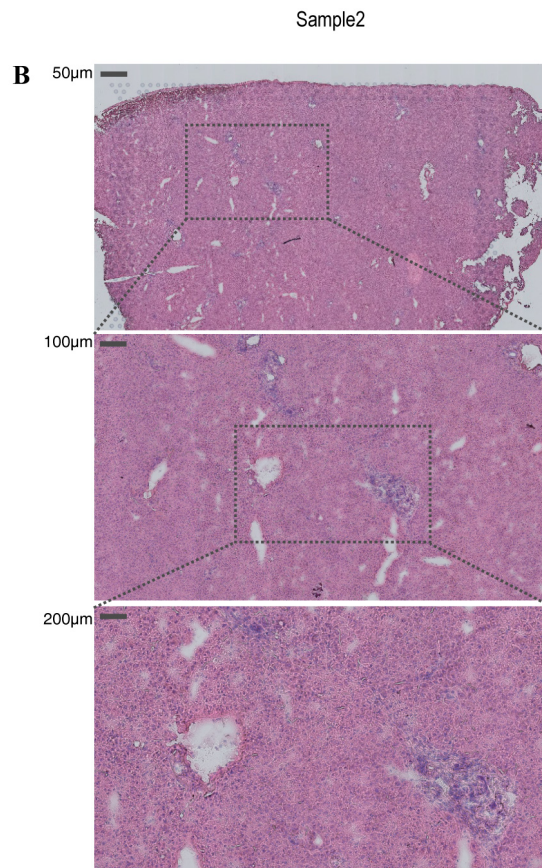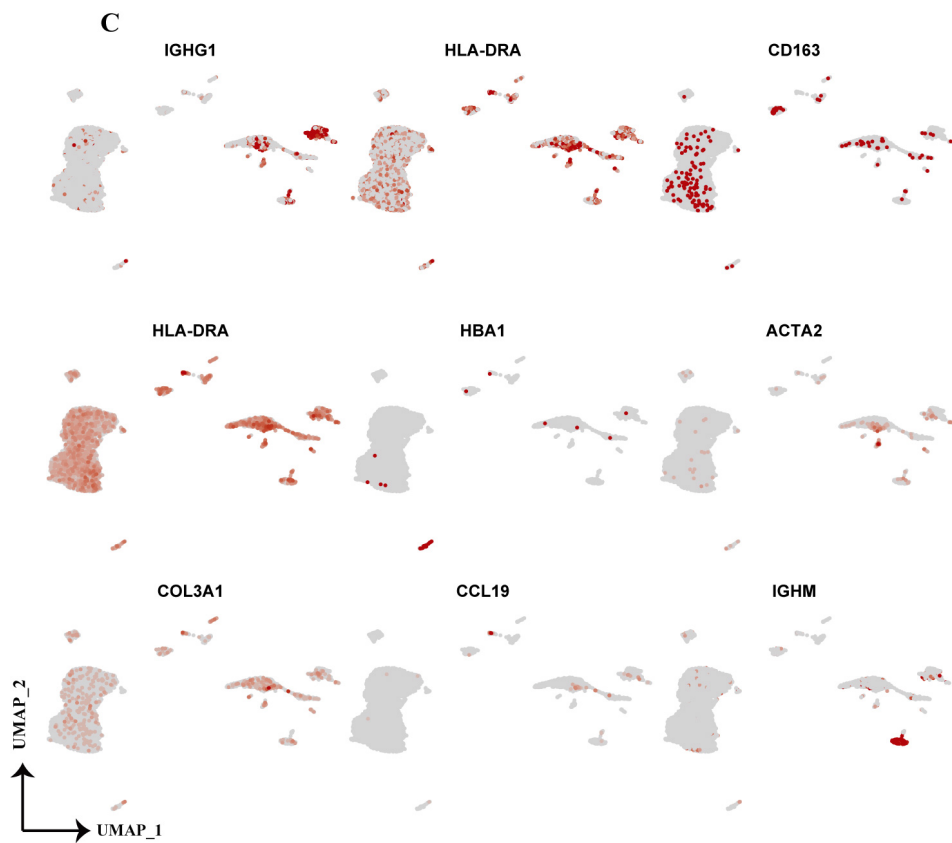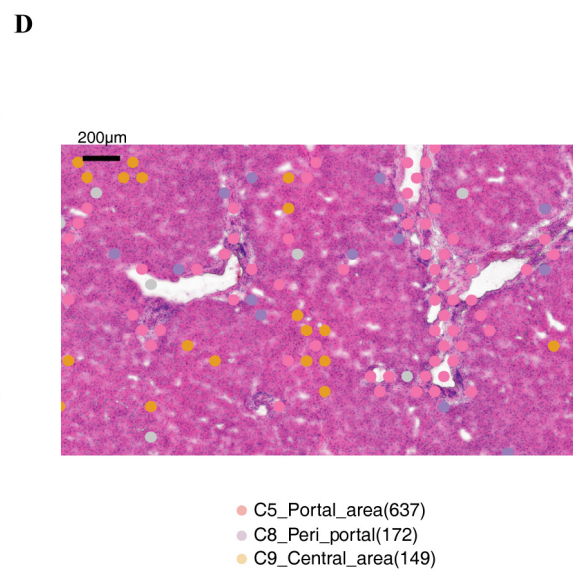

Supplement: Supplementary file 4 — Supplementary Figure 1 [file 41597_2022_1676_MOESM4_ESM.pdf]
