# Supplementary material for: Spatial transcriptome profiling of normal human liver
Source: Sci Data. 2022 Oct 19;9:633. doi: 10.1038/s41597-022-01676-w (PMC9581974; doi:10.1038/s41597-022-01676-w)
Supplement: Supplementary file 5 — Supplementary Figure 2 [file 41597_2022_1676_MOESM5_ESM.pdf]

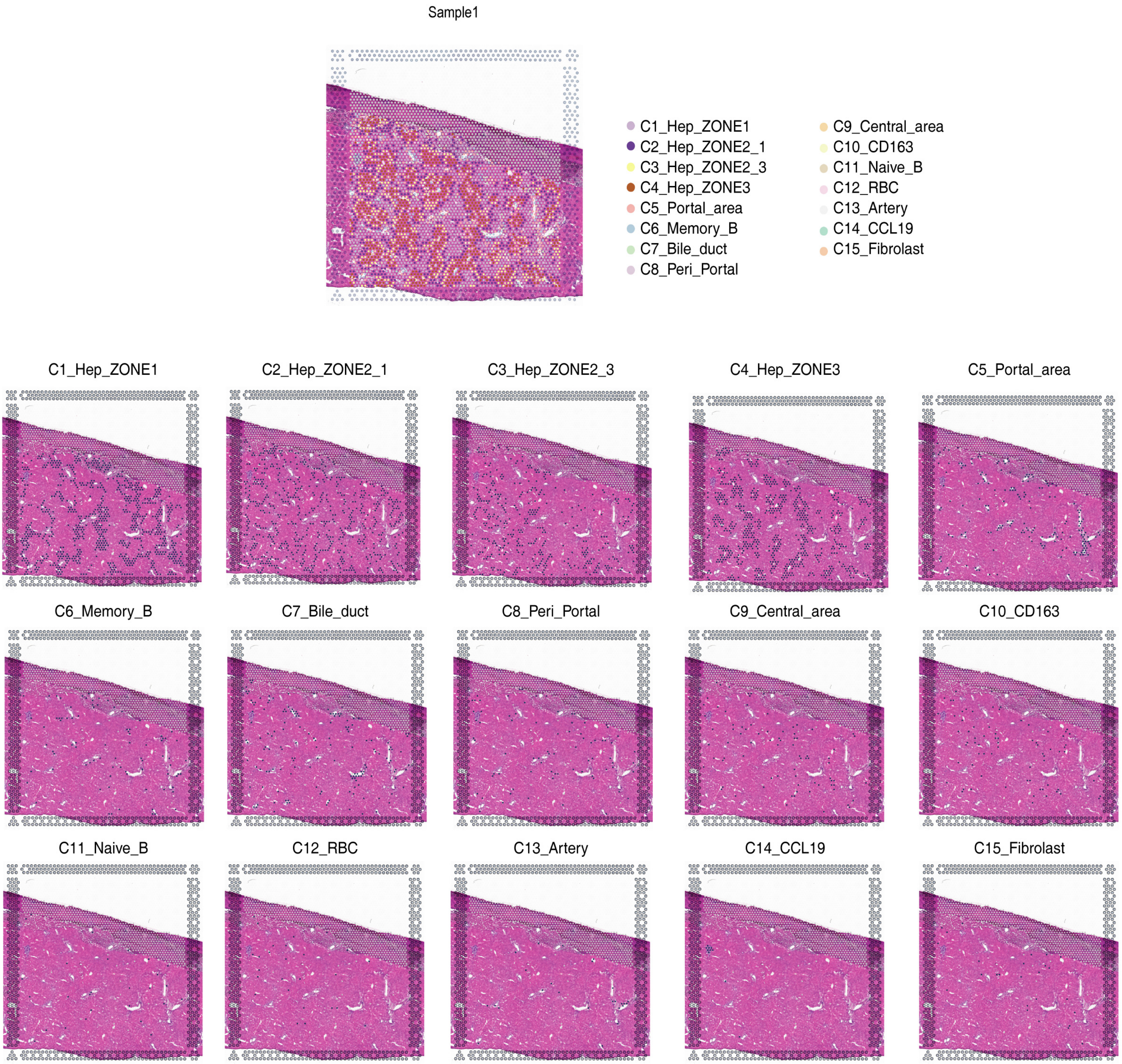
Sample1
C1_Hep_ZONE1
C2_Hep_ZONE2_1
C3_Hep_ZONE2_3
C4_Hep_ZONE3
C5_Portal_area
C6_Memory_B
C7_Bile_duct
C8_Peri_Portal
C9_Central_area
C10_CD163
C11_Naive_B
C12_RBC
C13_Artery
C14_CCL19
C15_Fibrolast
C1_Hep_ZONE1
C2_Hep_ZONE2_1
C3_Hep_ZONE2_3
C4_Hep_ZONE3
C5_Portal_area
C6_Memory_B
C7_Bile_duct
C8_Peri_Portal
C9_Central_area
C10_CD163
C11_Naive_B
C12_RBC
C13_Artery
C14_CCL19
C15_Fibrolast
